# An Application of the Patient Rule-Induction Method to Detect Clinically Meaningful Subgroups from Failed Phase III Clinical Trials

**DOI:** 10.23937/2469-5831/1510038

**Published:** 2021-06-28

**Authors:** Greg Dyson

**Affiliations:** Department of Oncology, Karmanos Cancer Institute, Wayne State University, Detroit MI, USA

**Keywords:** PRIM, Subgroup, Phase III, Survival, Classification

## Abstract

**Background:**

Phase III superiority clinical trials have negative results (new treatment is not statistically better than standard of care) due to a number of factors, including patient and disease heterogeneity. However, even a treatment regime that fails to show population-level clinical improvement will have a subgroup of patients that attain a measurable clinical benefit.

**Objective:**

The goal of this paper is to modify the Patient Rule-Induction Method to identify statistically significant subgroups, defined by clinical and/or demographic factors, of the clinical trial population where the experimental treatment performs better than the standard of care and better than observed in the entire clinical trial sample.

**Results:**

We illustrate this method using part A of the SUCCESS clinical trial, which showed no overall difference between treatment arms: HR (95% CI) = 0.97 (0.78, 1.20). Using PRIM, we identified one subgroup defined by the mutational profile in BRCA1 which resulted in a significant benefit for adding Gemcitabine to the standard treatment: HR (95% CI) = 0.59 (0.40, 0.87).

**Conclusion:**

This result demonstrates that useful information can be extracted from existing databases that could provide insight into why a phase III trial failed and assist in the design of future clinical trials involving the experimental treatment.

## Introduction

The drug development landscape is inundated with Phase III clinical trials where the primary efficacy endpoint was not met. Hay, et al., 2014 estimated that the likelihood of FDA approval for a drug already in Phase III development is 50%, which falls to 30% if we restrict to oncology [1]. Failed clinical trials have short- and long-term consequences for multiple parties. The patients who enrolled and took the experimental drug in the trial may have been exposed to an inferior treatment regime or experienced unnecessary toxicity without clinical benefit. They have also lost valuable time in their battle against their disease. Clinicians will not have an additional treatment option to manage the disease in their patients and may have treated patients with a sub- optimal treatment. Pharmaceutical companies will have invested years of research and multiple millions of dollars throughout the development of a drug that eventually fails to be approved for care. Most failed Phase III clinical trials are unsuccessful because efficacy of the new treatment was not established beyond the standard of care (SOC). Among the myriad of reasons (see Fogel, 2018 [2] for an extensive review) for the failure of these experimental treatments to become part of clinical practice, patient/disease heterogeneity stands out as a likely source. While every patient has a unique combination of genetic and environmental influences, historically the medical profession has treatment all patients as if they were the “average” patient even though every case cannot have experienced the same genetic and environmental exposures. More recently the medical profession has acknowledged that disease and patient heterogeneity is an important element of disease management as biomarker-driven clinical trials have become more common [3].

Even a drug that has an overall benefit and has been approved for use in clinical settings may in fact only benefit a subset of patients. Conversely, there are patients who may respond to a treatment that was an overall failure in a phase III clinical trial. Even if a treatment is effective for a subgroup of patients, it may not be clinically viable due to other reasons, including toxicity and cost. Determining the clinically feasible treatment most likely to have a positive outcome for an individual patient is one of the biggest Western medical challenges. As proscribing a unique treatment plan for each individual patient is currently unrealistic, the medical profession will often base treatment decisions on historical outcomes from subgroups of patients defined by variables and their values, e.g., the aggressiveness of treatment received is often based upon age, performance status, etc., utilizing prior knowledge to inform decisions. Ideally, data science techniques could be applied to determine which subgroup of patients should receive which treatment. Paradoxically though, we often cannot identify appropriate subgroups until studies have competed on a population-based sample or samples. Subgroups of clinical significance to investigators are sometimes pre-specified in the clinical protocol to ensure the research objectives are focused and to mitigate concerns about post-hoc data mining, multiple testing, and lack of power [4]. A clinical trial report will typically include a forest plot, which details how the experimental treatment performs relative to the SOC in pre- and non-pre-specific subgroups of interest via interaction effects. Analysis of combinations of subgroups defined by multiple variables are usually not performed. Non-pre-specified subgroup analyses are deemed exploratory and frequently not given the same scientific importance as pre-specified ones [4].

As classification methods are designed to create subgroups, they would seem to be ideal to tackle this problem. Recursive partitioning methods including Classification and Regression Trees [5] have been used to create subgroups for a variety of outcome types including ordinal, Gaussian and time to event. However, these approaches (see [6] for a brief description of both regression based and tree-based methods) have issues with overfitting, selection of the number of groups and stability of the resultant classification. In addition, they use only one variable at each step to define a subgroup; thus, these methods cannot detect non-additive effects defined by two or more variables. Regression-based models including linear, logistic, and Cox regression utilize all available samples and do not create subgroups, instead estimating an average effect for covariates included in the models. Interaction terms are used in these models to detect important subgroups but can have issues with empty/small N cells (including overfitting and lack of degrees of freedom) in higher dimensions and issues with multiple testing. Doove, et al. [7] provided a comparison of 5 recursive partitioning based methods for subgroup identification, including 3 methods [8–10] that focus on interaction effects and 2 methods [11,12] that focus on subgroups where the treated group outperforms the SOC. Bayesian approaches including the identification of credible intervals [13] utilize a similar objective as regression approaches: Interaction effects are modelled to identify subgroups where there is evidence of clinical benefit while accounting for multiple testing. A special issue on subgroup detection in clinical trials from the Journal of Biopharmaceutical Statistics [14] includes articles about the conceptual framework of subgroup detection and articles on different methodological approaches. These include a Bayesian approach utilizing a tree-based splitting process that results in predicted posterior probability of treatment effect [15] and a recursive partitioning based approach that screens for treatment by biomarker interaction effects to identify subgroups with a beneficial treatment effect with a multiple testing adjustment [16] (which is an extension of [12]). Other researchers have defined heuristics for evaluating a tree-based subgroup classification for a univariate response [6]. A statistical conundrum is that unless an exhaustive search of the space of all potential subgroups is explored important subgroups may be missed; but performing such a search may lead to overfitting and multiple testing concerns.

## Objectives

The objective of this paper is to use the Patient Rule-Induction Method (PRIM) [17] to identify subgroups of patients based on pre-treatment clinical and demographic characteristics where the experimental treatment is more effective than the SOC treatment and better than observed in the entire clinical trial cohort with a time-to-event outcome. Our previous enhancements [18–20] to the original PRIM algorithm [17] were developed in part to overcome some of the challenges posed from recursive partitioning methods, albeit for binary or continuous endpoints. Incorporating the combinational partitioning method (CPM) [21] allowed for non-additive effects to be used to define the subgroups in a way that does not fall victim to issues with empty/small N cells. The method also incorporates an element of statistical rigorousness (hypothesis testing via permutations) that is absent from most recursive partitioning procedures, which utilize cross validation or bootstrapping [22]. Other research groups have applied a PRIM based idea to analysis of data with survival endpoints [23–27]. Of particular importance to this manuscript is the paper by Chen, et al. [25], which had the same objective: To utilize a PRIM approach to identify a signature positive subgroup which will have better survival outcomes. As their approach was more theoretical, they did not show the terms or rules used to define the subgroups and set the minimum partition size to be 5% of the total sample size, using cross validation as a means to evaluate their technique. Our PRIM method is more translational than others proposed, as we aim to produce fewer subgroups of larger size so that the results can be simpler and more applicable in clinical practice and drug development. With a larger minimum subgroup size, our result should be more robust and have a greater chance of being validated, as we had previously shown in an analysis of heart disease incidence [28]. PRIM can readily include constraints on the outputted subgroups, including effect size and sample size. In this paper, we apply incorporate these concepts (CPM, hypothesis testing, subgroup constraints) into a differential survival analysis framework, which is novel for a PRIM approach. We will use PRIM to identify subsets of the patient population where the experimental treatment is more effective in terms of survival than the SOC treatment and then observed in the entire cohort. The results of this hypothesis-generating PRIM analysis can lead to further non-clinical and clinical investigations into the resultant subgroups.

## Methods

**Figure 1:**
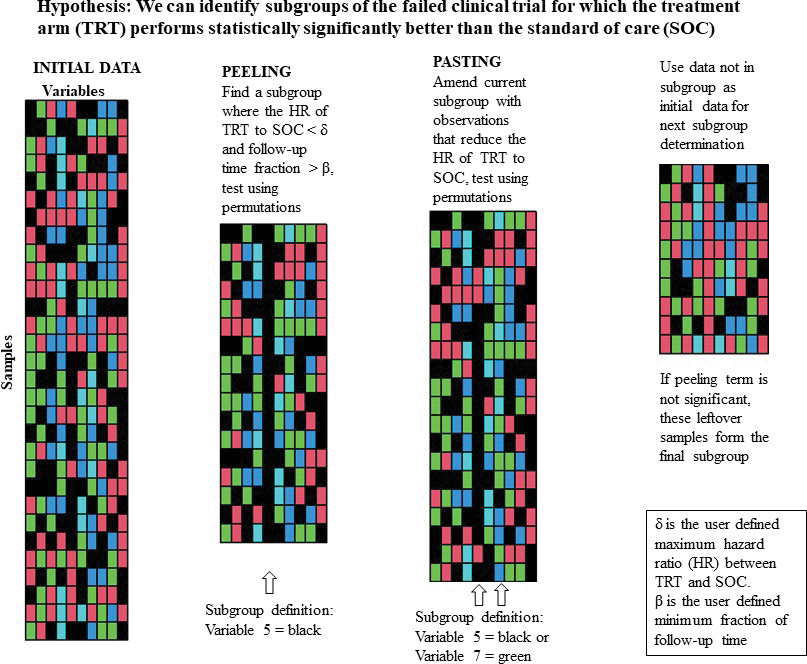
Graphic illustrating the PRIM algorithm including the peeling and pasting processes.

Mathematically, our objective is to determine which subgroup (if any) of observations of the input database has the smallest hazard ratio comparing the treatment (TRT) arm to the SOC arm. Multiple subgroups (or partitions) may be identified through repeated applications of the peeling and pasting process on smaller subsets of the input database (those observations not already assigned to a partition). Briefly, peeling is an iterative process that creates a partition by excluding individuals with particular values of predictor variables (creating a peeling term), while pasting iteratively amends individuals to the partition, also based upon values of predictor variables (creating a pasting term), after the peeling stage has been completed [18]. Once an observation is assigned to a partition, it cannot be assigned to another. Both processes create terms (defined by the values of one or more variables) to define the subsets of observations with a better clinical outcome. Each peeling and pasting term is tested for statistical significance using 2000 permutations of the input data set, keeping the response variables constant and shuffling the potential predictor variables. Figure 1 includes a graphic which gives an overview of the peeling and pasting processes.

Input variables used to define terms can be continuous, nominal or ordinal. While developing a mechanism for evaluating quantitative variables is feasible, to maintain the interpretability, we will convert continuous variables into ordinal ones. There are issues with using demarcation points (a BMI of 24.9 is normal while a BMI of 25.1 is overweight, even though the absolute difference in the numbers is small), but they are easier for a non-statistician to interpret. The demarcation points can either be inputted by the analyst or the program itself will create a four level ordinal variable: Greater than the mean +1 standard deviation (SD), between the mean and the mean +1 SD, between the mean −1 SD and the mean, and less than the mean −1 SD. PRIM will evaluate ordinal variables, starting at either end of the distribution, ensuring that adjacent categories are considered in the correct order, even when considering terms built using multiple variables. Ideally, most of the continuous variables would have demarcation points that can be defined from external sources so notions of bias can be mitigated. The incorporation of the CPM into the PRIM allows terms to be defined by non-additive combinations of predictor variables [19]. To ease the computational burden and increase the interpretability of the results, we restrict the algorithm to evaluate two predictor variables at a time to define each term that characterizes a partition.

This partitioning is controlled by two parameters: The minimum fraction of follow-up time (*β*) and the maximum hazard ratio between the TRT and SOC arms (δ). We use the fraction of follow up time rather than fraction of sample size as that is how the hazard ratio is indexed. The maximum hazard ratio defines a clinically meaningful difference between the treatment arms. To make the results more applicable to clinical practice and drug development, we evaluate *β*’s only in the range of 0.2 to 0.5, incremented by 0.005, forcing each partition to contain between 20% and 50% of the available samples. This will ensure that the resultant subgroups have sufficient coverage among the population of people with the given disease. The partitioning among those produced by each utilized *β* that results in the most significant Cox regression interaction term (with treatment group) is chosen as the outputted partitioning. We will use 75% of the hazard ratio observed in the entire cohort as the maximum hazard ratio, ensuring that the subgroups will result in at least a 25% improvement in the hazard of event as compared to the overall result. Taken together, these 2 constraints will ensure that created subgroups are adequately sized with a clinically meaningful effect size.

During the peeling process, all variables (one-at-a-time analysis) or possible pairs of variables (two-at-a-time analysis) are evaluated to identify a single term to define or further refine a partition. The term, among all potential terms that meets both the *β* and δ criteria, that has the smallest hazard ratio between the TRT and SOC arms is carried forward for hypothesis testing via permutations. If that term is statistically significant, the algorithm then tests another peeling term within the subset of observations already partitioned out. This continues until a peeling term is not significant; then pasting begins. During the pasting process, all variables (one-at-a-time analysis) or possible pairs of variables (two-at-a-time analysis) are evaluated to identify a single term to add to the current refine a partition, consisting of observations not already assigned to a partition. The term that results in the smallest hazard ratio between the TRT and SOC arms when amended to the observations already including in the partition via peeling and previous pasting steps is tested for significance. If it is significant, the algorithm adds those observations to the partition and looks for another pasting term. Once a pasting term is declared not statistically significant, the definition of that partition is complete. The peeling process will then begin again for the next partition, using only those observations not already in a previous partition. The algorithm stops whenever the first peeling term of any partition is not statistically significant.

## Data

### Simulation

A simulation study is undertaken to describe the operating characteristics of the method and explore the relationship between power and number of input variables. The simulation assumes that there are two treatment groups (treated and untreated), 1 biomarker of interest (not predictive of the response overall, predictive of response in the treated group, and not predictive of the response in the untreated group) and a variable number of null biomarkers. For the simulation, we assume that the hazard rate for the biomarker negative untreated is log(2)/10, biomarker negative treated is log(2)/8, and the biomarker positive untreated is log(2)/6. Both the biomarker positive treated hazard rate and the number of categorized random standard normal variables included in the analysis will be varied. The biomarker positive treated relative frequency is set at 30% for all simulations. The simulated treatment effect ranged from 0.90 to 1.25 in favor of the treated group, with 20 % of the simulations at the 0.90 hazard ratio showing an overall significant effect in favor of the treatment and less than 10% for all others. For each combination of biomarker positive median survival and number of null (no association with the outcome) continuous variables, 500 simulated clinical trials of 600 patients are created. The faux patients are randomized to treated or untreated arms with uniform accrual over 12 months and a maximum follow-up time of 12 months. Events time follow an exponential distribution, indexed by the above hazard rates and any patient alive without an event after 12 months post accrual is censored. We set the PRIM support parameter to 20% for all simulations and utilize a significance threshold of 0.10. The proportion out of 500 realizations that PRIM yields a significant result will be tabulated and plotted as described above.

### ACTG175 trial

We utilize an historical randomized clinical trial investigating AIDS treatments [29], available in the speff2trial package [30] in R to illustrate the utility of the CPM within a PRIM framework. The primary endpoint was a > 50% decline in the CD4 cell count, an event indicating progression to the AIDS, or death. The trial had four treatment arms: (0 = zidovudine, 1 = zidovudine and didanosine, 2 = zidovudine and zalcitabine, 3 = didanosine) and concluded that zidovudine alone was inferior to the other three treatment options [29]. We will compare (for illustrative purposes) arms 0 versus 1 < effective of didanosine given zidovudine > and arms 1 versus 3 < effective of zidovudine given didanosine >. The PRIM algorithm will choose the support parameter and variables will enter into the PRIM model two-at-a-time.

### SUCCESS trial

The SUCCESS trial [31] was designed to evaluate varying treatment regimens for high-risk breast cancer patients utilizing a two-stage randomization schema. The first randomization (denoted as SUCCESS-A) is study comparing disease-free survival of early primary breast cancer patients, who were randomized with standard of care (arm AB) or standard of care plus Gemcitabine (arm AA). The second randomization (SUCCESS-B) contrasted 2 or 5 year of adjuvant Zoledronic acid treatment. Through the dbGaP data portal (phs000547.v1.p1), we are able to access the data only relating to the first randomization, as that is all the data that was publicly released. The primary outcome is disease-free survival. Demographic information, tumor characteristics, and imputed SNPs were downloaded for all 3,322 participants. To keep the analysis simple and relevant to breast cancer, mutation information from only BRCA1 was used (139 SNPs). Further details on the study including a detailed description of the trial arms are located in the above reference and the dbGaP webpage (https://www.ncbi.nlm.nih.gov/projects/gap/cgi-bin/study.cgi?study_id=phs000547.v1.p1) for this study.

Since markers located in the same gene are in linkage disequilibrium (LD), we need to select out a subset of markers to construct a genetic profile that is unaffected by the amount of LD.

If there are too many highly correlated elements, they could dominate the clustering result and miss other structure. Other methods have been developed (see [32] for a review), but we utilize a simple clustering routine to choose the SNP subset. First, we calculate and utilize the pairwise r^2^ between all 139 SNPs as a distance metric in hierarchical clustering algorithm. Consequently, at each potential cut or split in the tree construction (starting at the root of the tree) we compute the minimum pairwise r^2^ for among each SNP pair contained within in each subgroup. Then we take the minimum of those minimums across all subgroups created by that split. Once the minimum of minimums reaches a certain threshold, say 0.95, we halt this process and declare that the split is optimal. This approach is different than other metric of determining an optimal split in a clustering routine (e.g., Silhouette width [33] or Dunn index [34]) in that we are not concerned about whether an object could be better grouped in another cluster or separation between clusters. Instead, the objective is to identify a clustering such that all elements within every cluster are highly correlated with the other elements in the same cluster.

The result will be *n* subgroup of SNPs where the r^2^ is at least 0.95 for all SNP pairs in each subgroup. As the selected threshold is 0.95, we anticipate many more clusters than a selection algorithm would identify, which likely would include singleton clusters. From each of the subgroups, we will then select a representative SNP (based on the number of non-missing elements) that will be utilized in the downstream analysis. Given this reduced set of SNPs, a hierarchical clustering of the patient samples (using the number of concordant genotype calls as a distance metric) will be produced and cut at the position where the minimum group size contains at least 100 samples to make the genetic information broadly applicable.

## Results

### Simulation study

**Figure 2:**
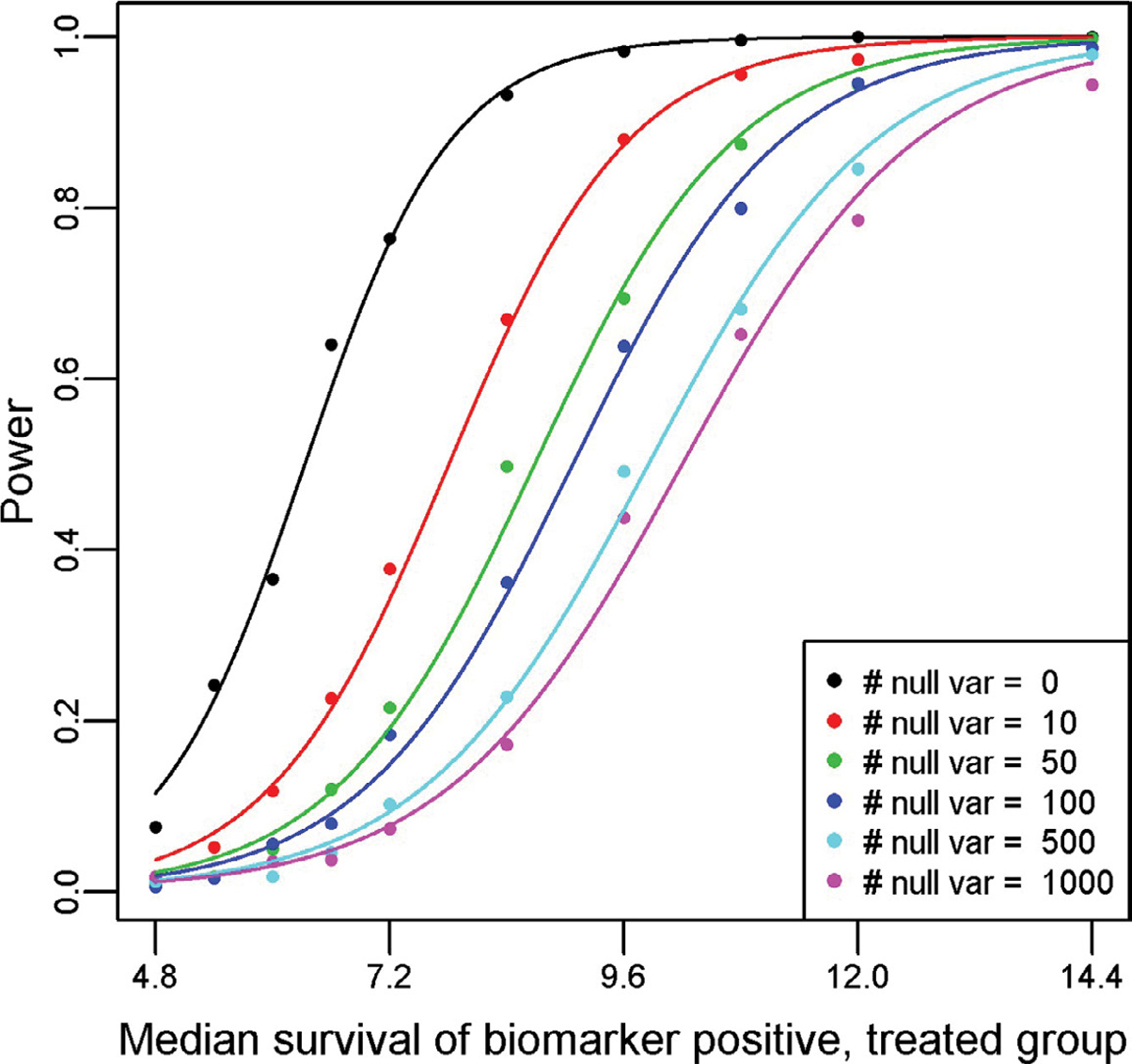
Power curves generated from a simulation study of the proposed method for a variety of null predictor variables and one true predictor variable. For the simulation, we assume that the hazard rate for the biomarker negative untreated is log(2)/10, biomarker negative treated is log(2)/8, and the biomarker positive untreated is log(2)/6. The median survival for the biomarker positive treated subgroup is plotted on the x-axis, with the resultant observed power from a PRIM analysis of detecting a significant effect from 500 simulated clinical trials of 600 patients on the y-axis.

Figure 2 displays the power curves from the simulation study, utilizing a Type I error rate of 0.10 for all simulations. Recall that we solve for the median survival for the biomarker positive, treated subgroup while fixing the median survival for the biomarker positive, untreated subgroup at 6 months; biomarker negative, treated subgroup at 8 months; and biomarker negative, untreated subgroup at 10 months. Thus, the hazard ratio for the biomarker negative subgroup (comparing treated relative to untreated) is 1.25. To achieve 80% power with no other variables, the median survival would have to be 7.37 in the biomarker positive treated arm as compared to 6 months in the biomarker positive untreated arm (hazard ratio = 0.814). Once we include 100 null variables, the median survival required rises to 10.57 months in the biomarker positive treated arm as compared to 6 months in the biomarker positive untreated arm (hazard ratio = 0.568). By employing a permutation-based significance testing, we account for multiple testing, reflected in the smaller hazard ratio required to achieve 80% power when the number of input predictors is increased. Therefore, it is imperative that only predictors of interest are included in the PRIM model (like any other statistical data analysis), so that the significance threshold when accounting for multiple testing is not too extreme. Pruning heavily confounded variables and collapsing variable categories will help keep the required threshold manageable. This will be an acute issue when analyzing genomic mutation datasets, as the LD between variants is not typically adjusted for in the modeling. These results informed our decision to collapse the SNP information from the SUCCESS-A trial into a single multi-level factor. Changing the support parameter or significance threshold used will change the values observed in the simulation, but the pattern and conclusion remains the same.

### ACTG175 trial

Table 1 displays a summarization of the variables that will be used in the construction of the PRIM subgroups. There was no difference in any baseline factor identified among the four groups using a Chi-square test. Overall the combination of zidovudine and didanosine was superior (p < 0.001) to zidovudine alone, hazard ratio (95% CI) = 0.49 (0.39, 0.63). PRIM identified one significant partition (partition 1, p-value = 0.08), defined by as [age ≥ 40 & Karnofsky score ≥ 90] that indicated a better survival outcome [HR (95% CI) = 0.23 (0.13, 0.42)] for patients randomized to the combination. The remainder partition (partition R, consisting of patients with age < 40 or Karnofsky score < 90) still showed a survival benefit (Cox regression p < 0.001) for the combination HR (95% CI) = 0.60 (0.46, 0.79). The combination of zidovudine and didanosine was not superior (p = 0.18) to didanosine alone overall, hazard ratio (95% CI) = 0.84 (0.65, 1.09). PRIM identified one significant partition (partition 1) that included a peeling (p = 0.09) and a pasting term (p = 0.08), defined as [white females and non-white males] or [homosexual activity and weight ≤ 60 kg] that indicated a better survival outcome for patients randomized to the combination. The subsetted out partition of samples had a HR (95% CI) of 0.36 (0.21, 0.62) in favor of the combination (p < 0.001). The remainder partition (partition R) still indicated no survival benefit (p = 0.42) for the combination versus didanosine alone, HR (95% CI) = 1.14 (0.88, 1.54).

### SUCCESS-A trial

Among the 3,322 patients enrolled in the SUCCESS-A trial, 3,312 had complete data for the progression endpoint, including both event status and time-to-progression. Following the algorithm outlined in the methods section, we utilized 33 BRCA1 SNPs out of the 139 (using 0.95 as the r^2^ threshold) to further analyze. There is strong LD among the selected SNPs because of the high r^2^ threshold used. Hierarchical clustering applied to these 33 SNPs resulted in the creation of nine mutually exclusive subgroups, containing at least 100 participants (the 10-subgroup split resulted in a subgroup with less than 100 members). In addition to the 9-level genetic variable, the traditional risk factors under consideration by the PRIM model are described in Table 2 on the 3,214 samples with complete data for these traditional risk factors. None of the variables were significantly different between the two treatment groups using a chi-squared test.

**Figure 3:**
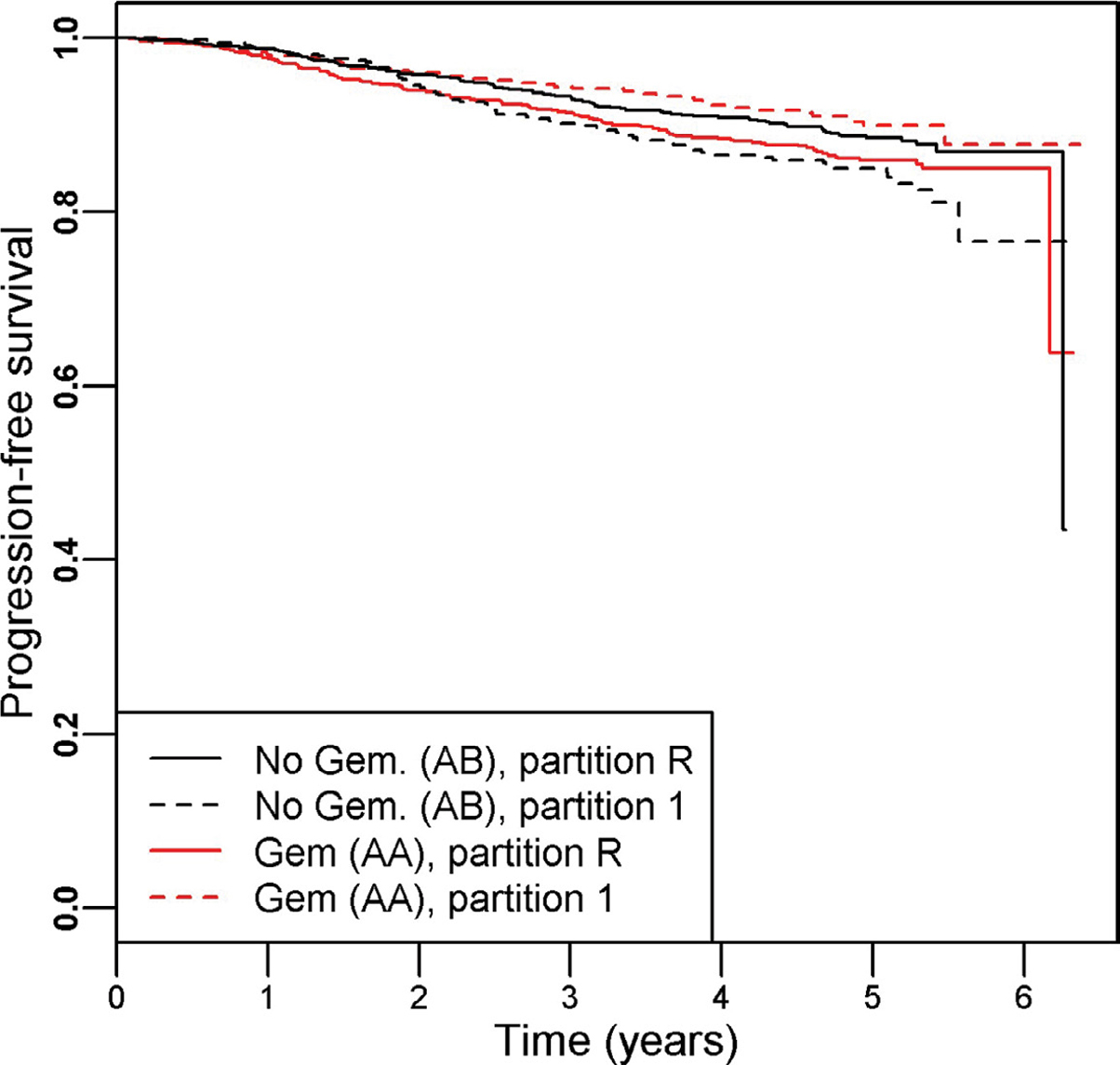
Kaplan-Meier curves illustrating the resultant partitions and treatment assignment relationship for the SUCCESS-A trial, which testing the inclusion of Gemcitabine to standard of care breast cancer therapy. Overall there was no difference in survival time between the study arms AA (with Gemcitabine) and AB (without Gemcitabine), HR (95% CI) = 0.97 (0.78, 1.20). Within partition 1 (defined as BRCA1 cluster id = 3 or 6) we find a significant improvement in survival for those randomized to the Gemcitabine arm: HR (95% CI) = 0.59 (0.40, 0.87). The remainder partition indicated no difference between the treatment arms: HR (95% CI) = 1.24 (0.96, 1.61).

There were 342 progression events and the hazard ratio (95% CI) of the patients treated with Gemcitabine (arm AA) versus those without (arm AB) was 0.97 (0.78, 1.20), indicating a lack of benefit of adding Gemcitabine. PRIM identified one significant partition (partition 1), defined by as [genomic cluster = 3 or 6] that indicated a better survival outcome for patients randomized to the Gemcitabine arm. The PRIM analysis used a support parameter of 0.32 and that term had a permutation-based p-value of 0.020. The subsetted-out samples had a HR (95% CI) of 0.59 (0.40, 0.87) in favor of the including Gemcitabine (Cox regression p = 0.007). The remainder partition (partition R) still indicated no survival difference (Cox regression p = 0.11) between the two arms, HR (95% CI) = 1.24 (0.96, 1.61). The Kaplan-Meier curves for the four groups are shown in Figure 3.

**Figure 4:**
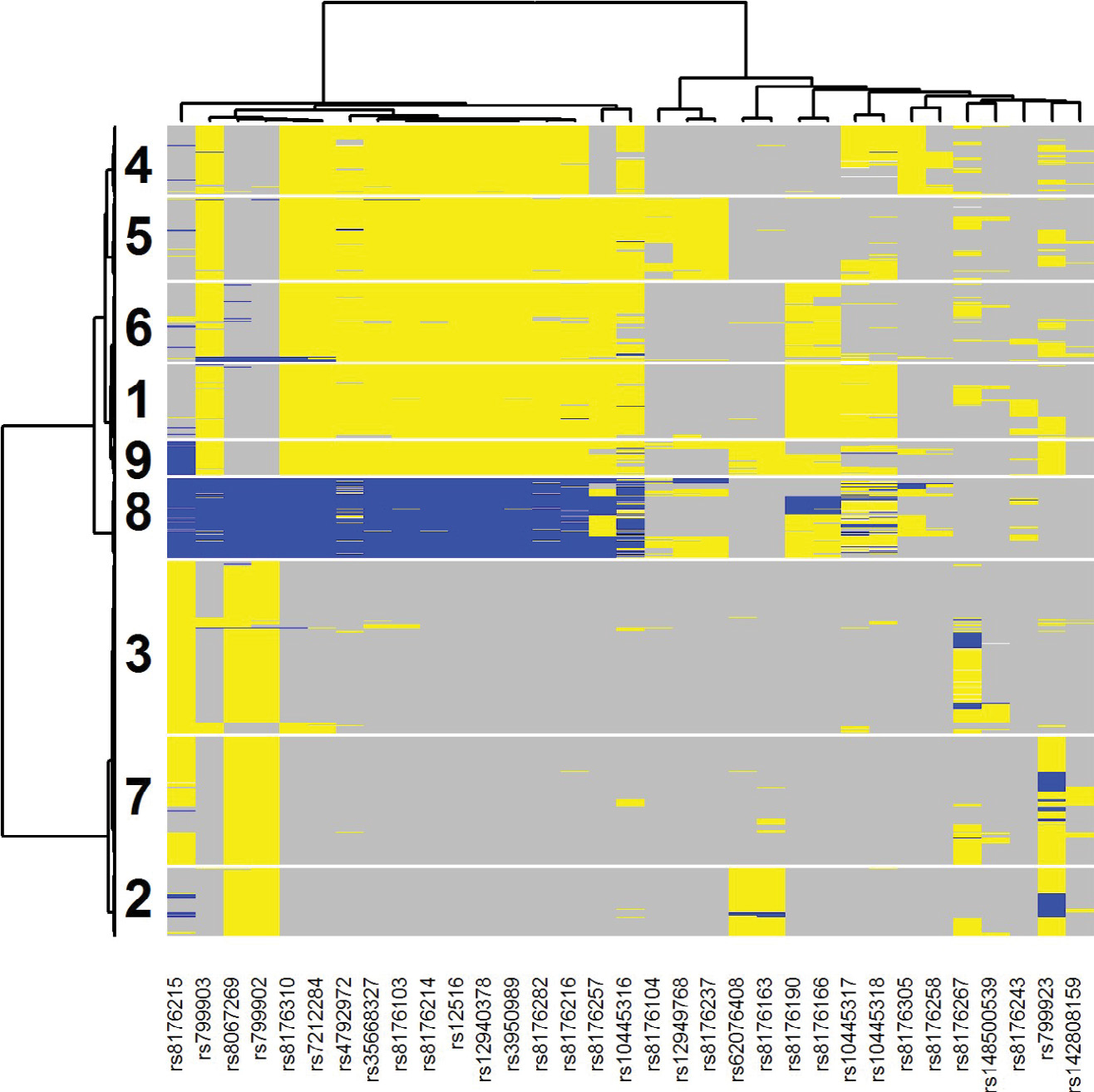
Two-way clustering diagram of the 33 selected SNPs from BRCA1 across all analyzed samples from the SUCCESS-A trial. Each SNP for each sample was color coded as gray (major homozygote [major allele based upon the study sample]), blue (heterozygote) or yellow (minor homozygote [major allele based upon the study sample]). Patients in clusters 3 and 6 were identified by PRIM as being indicative of better survival when randomized to the Gemcitabine arm.

Figure 4 illustrates the two-way clustering result for the 33 selected SNPs and all patients. Each SNP for each sample was color coded as gray (major homozygote [major allele based upon the study sample]), blue (heterozygote) or yellow (minor homozygote [major allele based upon the study sample]). Using this graph, we can select out five SNPs that can determine most of samples in clusters 3 and 6: rs3950989, rs799923, rs10445318, rs8176190, and rs8176215. If a patient is GG for both rs3950989 and rs799923 (cluster 3) or TT for rs10445318, rs8176190, and rs8176215 (cluster 6) then there is evidence that gemcitabine could be beneficial as part of their treatment [HR (95% CI) = 0.61 (0.41, 0.90)]. The patients with either of these two mutational profiles had a hazard ratio (95% CI) of 1.09 (0.86, 1.37) as compared to those patients with neither of the profiles, indicating that the interrelationship between treatment and genomics is driving the observed effect. Of the samples in the SUCCESS-A cohort, 969/3312 = 0.29 are in either of these two mutation profiles, illustrating the common nature of this suggestive subgroup.

## Discussion

Standard phase III clinical trial data analysis will typically include forest plots to evaluate whether variables (pre-defined or not) have a significant interaction effect with the treatment in predicting the primary endpoint (usually OS or PFS). These graphs have utility in suggesting future direction for researching the experimental treatment. The tool introduced in this paper represents an advanced data-driven data mining technique in the same vein as forest plots to generate subsequent hypotheses and experiments. The advantages of this method are that it can search over the entire space of variables, allows for non-additive two-variable terms can define the partitions, and adjusts for multiple testing via the permutation tests. While these subgroup analyses lack the power necessary to return a definitive answer, future clinical directions can be ascertained even from trials that failed to demonstrate efficacy of the primary endpoint. In the example presented in this paper, the genetic profile of BRCA1 appeared to affect the efficacy of Gemcitabine; future laboratory science and clinical trials involving that drug could work to tease out this relationship. A few disadvantages are present with this method: permutation analysis is computationally expensive, variable selection/pruning likely necessary (genomic component of the SUCCESS-A analysis) prior to analysis to avoid a too stringent multiple testing correction, and the resultant terms may lack readily discernible biological insights (ATCG175 analysis). We were able to overcome the computational costs for binary endpoints [20] by deriving analytic expressions for the null distribution. For Cox regression, no such expression exists, thus we are relegated to using permutations. Proportionality of the hazards is not checked when employing this method, although the resultant partitions can still be checked for proportionality. Utilizing restricted mean survival time rather than Cox regression as the survival analysis routine is a potential remedy if non-proportional hazards is expected to be an issue. This analysis demonstrates that when using the proper tools, useful information can be extracted from existing databases that could provide insight into why a phase III trial failed and assist in the design of future clinical trials involving the experimental treatment.

## Figures and Tables

**Table 1:** Sample description of the ACTG175 study variables considered by the PRIM algorithm. The arms are labeled as 0 = zidovudine, 1 = zidovudine and didanosine, 2 = zidovudine and zalcitabine, 3 = didanosine.

Treatment Group					
Variable	0 (n = 532)	1 (n = 522)	2 (n = 524)	3 (n = 561)	P-value

**Age**					0.984
≤ 30	166 (0.31)	164 (0.31)	164 (0.31)	177 (0.32)	
(30,40]	237 (0.45)	233 (0.45)	235 (0.45)	251 (0.45)	
(40,50]	102 (0.19)	101 (0.19)	95 (0.18)	111 (0.20)	
> 50	27 (0.05)	24 (0.05)	30 (0.06)	22 (0.04)	

**Weight (kg)**					0.749
≤ 60	44 (0.08)	63 (0.12)	58 (0.11)	66 (0.12)	
(60,70]	138 (0.26)	126 (0.24)	132 (0.25)	132 (0.24)	
(70,80]	177 (0.33)	175 (0.34)	172 (0.33)	192 (0.34)	
> 80	173 (0.33)	158 (0.30)	162 (0.31)	171 (0.30)	

**Hemophilia**					0.946
No	490 (0.92)	479 (0.92)	478 (0.91)	512 (0.91)	
Yes	42 (0.08)	43 (0.08)	46 (0.09)	49 (0.09)	

**Homosexual activity**					0.679
No	191 (0.36)	176 (0.34)	176 (0.34)	182 (0.32)	
Yes	341 (0.64)	346 (0.66)	348 (0.66)	379 (0.68)	

**Karnofsky score**					0.500
70	4 (0.01)	0 (0.00)	3 (0.01)	2 (0.00)	
80	17 (0.03)	22 (0.04)	18 (0.03)	23 (0.04)	
90	197 (0.37)	189 (0.36)	180 (0.34)	221 (0.39)	
100	314 (0.59)	311 (0.60)	323 (0.62)	315 (0.56)	

**Prior antiretroviral therapy**					0.350
No	516 (0.97)	513 (0.98)	511 (0.98)	552 (0.98)	
Yes	16 (0.03)	9 (0.02)	13 (0.02)	9 (0.02)	

**Race**					0.452
White	376 (0.71)	384 (0.74)	374 (0.71)	388 (0.69)	
Non-white	156 (0.29)	138 (0.26)	150 (0.29)	173 (0.31)	

**Gender**					0.708
Female	100 (0.19)	88 (0.17)	89 (0.17)	91 (0.16)	
Male	432 (0.81)	434 (0.83)	435 (0.83)	470 (0.84)	

					0.528
**Baseline CD4 count**	75 (0.14)	98 (0.19)	88 (0.17)	86 (0.15)	
≤ 15.3	182 (0.34)	175 (0.34)	175 (0.33)	197 (0.35)	
(15.3,18.4]	197 (0.37)	171 (0.33)	173 (0.33)	201 (0.36)	
(18.4,21.6]	78 (0.15)	78 (0.15)	88 (0.17)	77 (0.14)	
> 21.6					

**Baseline CD8 count**					0.813
≤ 6.32	72 (0.14)	79 (0.15)	69 (0.13)	88 (0.16)	
(6.32,6.79]	197 (0.37)	173 (0.33)	181 (0.35)	192 (0.34)	
(6.79,7.26]	190 (0.36)	183 (0.35)	195 (0.37)	203 (0.36)	
> 7.26	73 (0.14)	87 (0.17)	79 (0.15)	78 (0.14)	

**Table 2:** Sample description of the SUCCESS-A study variables considered by the PRIM algorithm, including the BRCA1-defined cluster id. Patients were randomized to either standard of care (arm AB) or standard of care plus Gemcitabine (arm AA).

Variable	Treatment		
	AA (n = 1590)	AB (n = 1624)	P-value

**ER status**			0.411
Positive	1086 (0.68)	1132 (0.70)	
Negative	504 (0.32)	492 (0.30)	

**HER2 status**			0.188
Positive	509 (0.32)	484 (0.30)	
Negative	1081 (0.68)	1140 (0.70)	

**PR status**			0.456
Positive	1003 (0.63)	1046 (0.64)	
Negative	587 (0.37)	578 (0.36)	

**MenoDause status**			0.334
Premenopausal	667 (0.42)	653 (0.40)	
Postmenopausal	923 (0.58)	971 (0.60)	

**Grade**			0.451
G1 (well differentiated)	81 (0.05)	68 (0.04)	
G2 (moderately differentiated)	763 (0.48)	778 (0.48)	
G3 (poorly differentiated)	746 (0.47)	778 (0.48)	

**T stage**			0.823
pT0	1 (0.00)	0 (0.00)	
pT1	657 (0.41)	672 (0.41)	
PT2	830 (0.52)	838 (0.52)	
pT3	81 (0.05)	90 (0.06)	
pT4	21 (0.01)	24 (0.01)	

**N stage**			0.487
pN0	570 (0.36)	561 (0.35)	
pN1	697 (0.44)	747 (0.46)	
pN2	229 (0.14)	213 (0.13)	
pN3	94 (0.06)	103 (0.06)	

**Tumor type**			0.899
Invasive ductal	1252 (0.79)	1278 (0.79)	
Invasive lobular	165 (0.10)	175 (0.11)	
Other invasive epithelial breast cancer	173 (0.11)	171 (0.11)	

**Age categorized**			0.583
(−Inf,50]	654 (0.41)	639 (0.39)	
(50,65]	693 (0.44)	727 (0.45)	
(65, Inf]	243 (0.15)	258 (0.16)	

**BMI categorized**			0.387
(−Inf,25]	739 (0.46)	771 (0.47)	
(25,30]	537 (0.34)	513 (0.32)	
(30, Inf]	314 (0.20)	340 (0.21)	

**CLUSTER ID**			0.538
1	144 (0.09)	159 (0.10)	
2	150 (0.09)	123 (0.08)	
3	353 (0.22)	345 (0.21)	
4	139 (0.09)	142 (0.09)	
5	156 (0.10)	185 (0.11)	
6	169 (0.11)	161 (0.10)	
7	254 (0.16)	265 (0.16)	
8	155 (0.10)	172 (0.11)	
9	70 (0.04)	72 (0.04)	
